# Comparative outcomes of two different styles of mental health practice

**DOI:** 10.1192/j.eurpsy.2025.1609

**Published:** 2025-08-26

**Authors:** L. Mehl-Madrona, B. Mainguy

**Affiliations:** 1 Native Studies, University of Maine, Orono; 2 Psychiatry Residency, Northern Light Acadia; 3 Wabanaki Health and Wellness, Bangor, United States

## Abstract

**Introduction:**

An opportunity arose to compare the outcomes of patients of one psychiatrist in two different clinical settings – a community mental health center CMHC) in which the psychiatrist saw people on average for 15 minutes every 6 weeks (range 4 to 12 weeks) and a community clinic setting (CCS) in which the psychiatrist controlled the time allotted per patients and the frequency of visits. We assumed that the psychiatrist’s beliefs, attitudes, and style of practice did not change between the two settings except as influenced by time constraints. Psychotherapy was provided by social workers in both settings, with an average of 45 minutes every 3 weeks in the CMHC and 40 minutes every week in the CCS. Three optional groups existed in the CCS compared to one in the CMHC. New patients received a 30 minute evaluation in the CMHC and a 60 minute evaluation in the CCS.

**Objectives:**

To compare the dominant style of practice in the United States with an older style of practice in which psychiatrists spent more time with clients.

**Methods:**

The psychiatrist administered the MYMOP2 (My Medical Outcome Profile, version 2) and the Brief Psychiatric Rating Scale (BPRS) to all patients at baseline in both settings. The MYMOP2 was repeated monthly (or at the next visit in the CMHC) and the BPRS at intervals of every three months. The study lasted two years and the average length of follow-up was 31 weeks in the CMHC and 49 weeks in the CCS, which was statistically significant.

**Results:**

No statistically significant differences appeared in demographic variables. Percent funded by Medicaid, Medicare, other insurance, gender, and age distribution were the same in both settings. Clinical improvement was not observed among patients on average on both measures in the CMHC. Clinical improvement was observed on both measures in the CCS (MYMOP-2; p < 0.01 on worst symptom; BPRS, p < 0.01). The CMHC showed higher profits than the CCS. Time spent per patient was statistically significantly greater in the CCS (p < 0.01).

**Conclusions:**

Increased opportunity for contact and relationship with the psychiatrist may play a greater role than assumed by the biomedical model. A public health question arises in relation to models for provision of care that are more profitable but less health effective.

**Disclosure of Interest:**

None Declared

